# Administration of bisphosphonate for hypercalcemia associated with oral cancer

**DOI:** 10.1186/1746-160X-2-9

**Published:** 2006-04-10

**Authors:** Kojiro Onizawa, Hiroshi Yoshida

**Affiliations:** 1 Oral and Maxillofacial Surgery, Doctoral Program in Functional and Regulatory Medical Sciences, Graduate School of Comprehensive Human Sciences, University of Tsukuba, Japan

## Abstract

**Background:**

The efficacy of treating hypercalcemia with bisphosphonate (BP) in patients with advanced oral cancer has not been fully investigated. This retrospective study evaluated the clinical course of hypercalcemic patients with and without BP treatment.

**Methods:**

Sixteen hypercalcemic patients, most of whom had uncontrollable locoregional lesions and lung metastases, were studied. Nine patients had been given BP, and the rest had not.

**Results:**

There were significant differences in age and serum ALT between the BP-treated and -untreated groups. The first administration of BP effectively and safely decreased the serum calcium level, but repeated administrations were less effective. Although the patients treated with BP survived significantly longer than the untreated subjects, the difference of the median was only about 2 weeks.

**Conclusion:**

The occurrence of hypercalcemia in oral cancer patients apparently implies an extremely poor prognosis, and long-term survival cannot be expected, even with BP treatment.

## Background

Cancer-associated hypercalcemia (CAH) frequently occurs in patients with advanced oral cancer and indicates that the patients have entered the terminal stage of the disease [1]. Increased serum calcium (Ca) levels induce symptoms in the gastrointestinal, kidney, and central nervous systems [2], reducing the patients' quality of life (QOL). Although CAH cannot be adequately resolved without controlling tumor progression, the administration of bisphosphonate (BP) has been reported to effectively decrease the serum Ca level, and improve QOL [2-4]. Especially in the case of patients with a slow-growing tumor, such as breast cancer, BP can significantly control pain caused by bone metastasis [5]. Accordingly, BP administration might be useful to improve the QOL of CAH patients in whom long-term survival is anticipated. However, advanced oral cancers usually progress rapidly, and the average survival time after the occurrence of CAH is approximately 1 or 2 months [1,6]; thus, BP treatment might not be helpful for all CAH patients with advanced oral cancer. However, there have been few reports on the clinical effects of giving BP to oral cancer patients with CAH, so its potential usefulness is uncertain.

Here we retrospectively investigated the clinical course of CAH patients with advanced oral cancer who were or were not treated for CAH with BP.

## Methods

A retrospective analysis was conducted of all patients who received a diagnosis of hypercalcemia associated with oral cancer at the Department of Oral and Maxillofacial Surgery of University of Tsukuba Hospital, between 1995 and 2004. Hypercalcemia was defined as a serum Ca level of higher than 11 mg/dl, which is the upper limit of the normal range in healthy individuals, in tests performed at the clinical laboratory. The value for the serum Ca level was corrected against the value of the serum albumin level [7]. The decision to treat with BP was determined by state of tumor advancement, symptoms caused by CAH and general condition.

Patient characteristics such as the primary tumor site, stage of tumor progression, general condition, laboratory test values at the diagnosis of CAH, and the administration of anti-tumor and anti-CAH agents were determined by reviewing medical records. The serum Ca levels assayed before and after the BP administration were examined to evaluate the effect of the BP treatment. The laboratory findings from before BP administration were compared between patients treated with BP (BP group) and those that did not receive BP (non-BP group). Survival time was calculated as the period from the occurrence of hypercalcemia to death, and the factors influencing the survival time were evaluated.

The Mann-Whitney test was applied to compare the influence of age and laboratory findings on the occurrence of CAH in the BP and non-BP groups. Survival time after the occurrence of CAH was also analyzed statistically using the Mann-Whitney test within each set of clinical and laboratory characteristics for all patients. Differences with a p value of less than 0.05 were considered statistically significant.

## Results

### 1. Characteristics of patients at the diagnosis of CAH

The 16 subjects were 10 men and 6 women, with CAH and advanced oral cancer, and their age ranged from 24 to 84 years, with a mean of 60.4 years. The primary sites of oral cancer were the tongue in 6 patients, the lower gingiva in 2, and the maxillary sinus in 2, and 1 each had as the primary site the floor of mouth, the buccal mucosa, the upper gingiva, and the oropharynx. Of the remaining 2 patients, one had simultaneously duplicated oral cancers of the lower and upper gingivas, and another had metachronous multiple oral cancers of the lower gingiva, buccal mucosa, and tongue. The time from the initial diagnosis of oral cancer to the occurrence of CAH ranged from 3 to 81 months, with a median of 11.5 months. The value of parathyroid hormone related protein (PTHrP), which was measured in 7 patients, ranged from 103 to 475 pmol/l, with a median of 160.4 pmol/l; these values were higher than the 16.2 to 64.7 pmol/l normal range found at our hospital.

Of the 16 patients, 12 had uncontrollable head and neck lesions, lung or pleural metastases were observed in 14 (87.5%), and 7 had bone metastases (43.8%, Table 1).

**Table 1:** Tumor progression in CAH patients at diagnosis

Lesion	Patients (%)
Uncontrollable locoregional lesions	12 (75.0)
Distant metastases
Lung, pleura	14 (87.5)
Bone	7 (43.8)
Skin	3 (18.8)
Liver	2 (12.5)
Kidney	2 (12.5)

### 2. Treatment for CAH

Nine of the 16 patients were treated with BPs for CAH (BP group). The remaining 7 patients did not undergo a specific therapy for CAH (non-BP group). On average, the BP group was significantly younger and showed significantly lower alanine aminotransferase (ALT) levels than the non-BP group, but there were no statistical differences in the other laboratory variables (Table 2).

**Table 2:** Comparison of BP-treated and non-BP-treated patient groups

Characteristics	BP group (n = 9)	non-BP group (n = 7)	p value*
	median (min.~max.)	median (min.~max.)	
Age	50(24~73)	76 (58~84)	0.0050
WBC (x1000/m^3^)	7.1 (6.1~16.4)	8.4 (4.1~35.8)	0.958
Hb (g/dl)	10.3 (7.3~14.5)	11.2 (9.2~14.4)	0.397
Plt (x10000/m^3^)	32.9 (152~742)	27.8 (192~438)	0.315
AST (IU/dl)	17 (11~87)	26 (13~106)	0.290
ALT (IU/dl)	14 (6~129)	48 (9~267)	0.050
LDH (IU/dl)	173 (112~487)	287 (137~408)	0.596
ALP (IU/dl)	269 (184~721)	322 (171~867)	0.427
Cre (mg/dl)	0.8 (0.5~1.0)	0.8 (0.3~0.9)	0.871
CRP (mg/dl)	3.3 (0.7~19)	4.7 (1.4~13.8)	0.186

*: Mann-Whitney test

The BP group received BP in the form of pamidronate (30 mg, 3 patients) or incadronate (10 mg, 6 patients). The medications were given 1 to 5 times, with a mean of 2.7 times. The median serum Ca level before the initial administration was 12.9 mg/dl, ranging from 12.2 to 16 mg/dl, and after the administration the median Ca level decreased to 10.6 mg/dl, ranging from 9.6 to 12.2 mg/dl. The median amount of decrease was 2.2 mg/dl. The time from the initiation of treatment to the maximum effect on Ca levels ranged from 4 to 7 days, with a median of 5 days. Six patients received two or more treatments, with a median interval of 14 days. The median decreases in serum Ca levels after the 2nd and 3rd administrations were 1.5 mg/dl and 0.7 mg/dl, respectively (Table 3). Most patients showed improved consciousness in association with the decrease of serum Ca level caused by BP administration. None of the patients showed a serious adverse effect from the treatment.

**Table 3:** Serum Ca control with repeated administrations of BP

	mean ± s.d.	median	min. ~max.
1^st ^administration (n = 9)			
pre Ca (mg/dl)	13.4 ± 1.2	12.9	12.2 ~16.0
post lowest Ca (mg/dl)	10.7 ± 0.8	10.6	9.6 ~12.2
Ca decrease (mg/dl)	2.7 ± 1.5	2.2	1.6 ~6.1
2^nd ^administration (n = 6)			
pre Ca (mg/dl)	13.0 ± 1.2	12.5	11.8 ~14.5
post lowest Ca (mg/dl)	11.4 ± 0.9	11.2	10.5 ~13.0
Ca decrease (mg/dl)	1.6 ± 0.7	1.5	0.7 ~2.7
3^rd ^administration (n = 4)			
pre Ca (mg/dl)	13.3 ± 1.1	13.1	11.8 ~14.7
post lowest Ca (mg/dl)	12.8 ± 2.3	11.8	10.7 ~16.0
Ca decrease (mg/dl)	0.5 ± 1.5	0.7	-1.9 ~2.2

### 3. Survival time after diagnosis of CAH

The serum Ca level measured within 2 days of death ranged from 9.6 to 15.7 mg/dl, with a mean value of 12.5 mg/dl. Most patients died of respiratory insufficiency caused by the progression of pulmonary metastases. The survival time from the diagnosis of CAH in the 16 patients ranged 3 to 152 days, with a median duration of 31 days. There was no significant difference in the survival time associated with any of the clinical and laboratory characteristics except the administration of BP: The patients in the BP group lived significantly longer than those in the non-BP group (Table 4).

**Table 4:** Days survived after CAH

Characteristics	median (min. ~max.)	p value*
Gender		
Male (n = 10)	38 (13 ~152)	0.117
Female (n = 6)	22 (3 ~46)	
Age		
60 > (n = 8)	40.5 (13 ~152)	0.138
60 ≦(n = 8)	27 (3 ~69)	
Primary site		
Tongue (n = 6)	32 (13 ~46)	0.479
Others (n = 10)	31 (3 ~152)	
Time from tumor diagnosis to CAH		
Within 1 year (n = 9)	43 (10~152)	0.139
Over 1 year (n = 7)	27 (3~46)	
WBC (x 1000/m^3^) at diagnosis of CAH		
10 > (n = 10)	38 (17 ~152)	0.103
10 ≦(n = 6)	20 (3 ~46)	
Hb(g/dl) at diagnosis of CAH		
10.9 > (n = 8)	41.5 (17 ~152)	0.092
10.9 ≦(n = 8)	22 (3 ~46)	
Serum ALP (IU/dl) at diagnosis of CAH		
300 > (n = 6)	51 (27 ~152)	0.118
300 ≦(n = 10)	28 (3 ~46)	
Serum CRP (mg/dl) at diagnosis of CAH		
4.0 > (n = 8)	38.5 (27 ~152)	0.058
4.0 ≦(n = 8)	22 (3 ~46)	
Treatment with BP		
Yes (n = 9)	44 (13 ~152)	0.015
No (n = 7)	27 (3 ~43)	
Treatment with Anticancer agent		
Yes (n = 5)	33 (17 ~69)	0.868
No (n = 11)	29 (3 ~152)	

*: Mann-Whitney test

## Discussion

CAH is classified as a humoral hypercalcemia of malignancy or local osteolytic types. CAH occurring in patients with advanced, uncontrollable oral cancer is usually humoral hypercalcemia, and is associated with a rise in the PTHrP level [1,6]. In the current study, a rise in the PTHrP level was observed in all 7 patients in whom the level was measured, and fewer than half of the patients had evidence of local bone destruction or bone metastasis. Consequently, in this study the CAH of the patients was most likely not local osteolytic CAH but humoral CAH.

The administration of BP for CAH is considered to be a standard therapy. Pamidronate and incadronate are reported to induce a decrease in the serum Ca level of about 2 mg/dl. The time from the initiation of BP administration to the occurrence of the maximum effect is 4 days, and the effect continues for two weeks [8]. The present study showed a decrease in the serum Ca level of about 2 mg/dl after the first administration, a maximal effect at day 5, and an effective duration of 2 weeks, as previously reported [8]. Repeated administrations were less effective, but the usefulness and safety of BP administration was confirmed for CAH patients with advanced oral cancer.

The administration of BP has not been indicated for all patients with CAH. Heath stated that not all patients with severe hypercalcemia should be treated, and that the decision should depend on the recent QOL of patients, current symptoms, and the prospect of further treatment for the malignancy [9]. Ling et al. reported that the primary aim of the treatment should be symptom control, and that the benefit of the treatment should be questioned for patients with neither a further indication for anti-cancer therapy nor symptoms caused by CAH [10]. Lamy et al. claimed that antihypercalcemic treatment should be guided by the severity of the hypercalcemia (> 3.00 mM/L, 12 mg/dl), not by the symptoms [4]. We decided the administration of BP, in consideration of serum Ca level of higher than 12 mg/dl, stages of the tumor progression, symptoms caused by hypercalcemia and general condition at the diagnosis of CAH. As the results, there was significant difference in age and ALT level between BP and non-BP groups. The difference in age was probably attributable to the fact that older patients and their families often prefer that no further treatment be given at the terminal stage, as they desire an earlier release from the suffering caused by an incurable disease. Younger patients and their families frequently choose additional intervention, since they wish the patient to have as long a life as possible. The difference in the ALT level also implies that the decision to give BP took into consideration the degree of liver dysfunction.

BP administration has been reported to prolong survival time [1,6]. The present study also showed a significant prolongation of survival time in the BP group compared with the non-BP group, although it was not a randomized control study and included some bias. As a raised serum level of PTHrP is reported to indicate a reduced hypocalcemic response to BP and an extremely poor prognosis [11], the anticipated survival time after the occurrence of CAH is 1 or 2 months in oral cancer, even if BP is given. CAH patients with oral cancer commonly have uncontrollable locoregional lesions causing serious cosmetic, functional, and social disturbances, which continue to deteriorate until their death. These disturbances might be easier to accept with the low level of consciousness caused by hypercalcemia. Consequently, whether to treat CAH with BP should be determined individually on the basis of a patient's physical and mental status and likely survival time, even if BP administration is effective and safe for controlling the serum Ca level.

## Conclusion

The occurrence of hypercalcemia in oral cancer patients apparently implies an extremely poor prognosis, and long-term survival cannot be expected, even with BP treatment. Whether to treat CAH with BP should be determined individually on the basis of a patient's physical and mental status and likely survival time.

## Competing interests

The author(s) declare that they have no competing interests.

## Authors' contributions

KO designed the study and analyzed the data. KO and HY contributed to writing the paper. All authors read and approved the final manuscript.
